# Significance of tumor infiltrating granulocytes and neutrophil extracellular traps in colorectal cancer

**DOI:** 10.1038/s41416-026-03409-x

**Published:** 2026-04-07

**Authors:** Oskari Rahkola, Anne Tuomisto, Hanna Elomaa, Henna Karjalainen, Ville K. Äijälä, Meeri Kastinen, Vilja V. Tapiainen, Akseli Kehusmaa, Aino Ojanperä, Vesa-Matti Pohjanen, Maarit Ahtiainen, Olli Helminen, Erkki-Ville Wirta, Jukka Rintala, Sanna Meriläinen, Raila Aro, Reetta Häivälä, Juha Saarnio, Tero Rautio, Toni T. Seppälä, Jan Böhm, Jukka-Pekka Mecklin, Markus J. Mäkinen, Juha P. Väyrynen, Päivi Sirniö

**Affiliations:** 1 Department of Pathology, Translational Medicine Research Unit, University of Oulu, Medical Research Center Oulu and Oulu University Hospital, Oulu, Finland; 2 Department of Education and Research, Well Being Services County of Central Finland, Jyväskylä, Finland; 3 Central Finland Biobank, Hospital Nova of Central Finland, Well Being Services County of Central Finland, Jyväskylä, Finland; 4 Department of Gastroenterology and Alimentary Tract Surgery, Tampere University Hospital, Tampere, Finland; 5 Faculty of Medicine and Health Technology, Tampere University and Tays Cancer Centre, Tampere University Hospital, Tampere, Finland; 6 Department of Gastrointestinal Surgery, Helsinki University Central Hospital, University of Helsinki, Helsinki, Finland; 7 Applied Tumor Genomics, Research Program Unit, University of Helsinki, Helsinki, Finland; 8 Department of Pathology, Hospital Nova of Central Finland, Well Being Services County of Central Finland, Jyväskylä, Finland; 9 Faculty of Sport and Health Sciences, University of Jyväskylä, Jyväskylä, Finland

**Keywords:** Tumour immunology, Neutrophils, Cytokines, Colorectal cancer

## Abstract

**Background:**

Neutrophils are the most abundant granulocytes in the tumor microenvironment and exert both pro- and anti-cancer effects. Activated neutrophils can release neutrophil extracellular traps (NETs) that have been proposed to promote tumor progression and metastasis. We aimed to clarify the significance of NETs and granulocytes in colorectal cancer.

**Methods:**

Our study population consisted of three independent colorectal cancer cohorts (*N* = 1927). We identified NETs showing positivity for citrullinated histone H3 (Cit-H3) and granulocytes expressing CD66b (CEACAM8) with multiplex immunohistochemistry and used digital image analysis and machine learning tools to calculate their densities in tumor samples. Associations between NET and granulocyte densities with clinicopathologic characteristics, tumor-infiltrating immune cells, prognosis, and systemic inflammation markers were examined.

**Results:**

The densities of Cit-H3^+^ NETs positively correlated with macrophage densities and mesenteric serum levels of CX3CL1 and ANGPT2, but were not associated with survival. Higher CD66b^+^ granulocyte density was associated with longer colorectal cancer-specific survival independent of conventional prognostic parameters. In the largest cohort (*N* = 1090), multivariable HR for high vs. low CD66b^+^ granulocyte density was 0.53 (95%CI 0.38–0.73, *P*_Trend_ < 0.001).

**Conclusion:**

Our findings indicate that while neutrophil infiltration is associated with favorable colorectal cancer outcomes, the presence of intratumoral Cit-H3^+^ NETs does not predict survival.

## Introduction

Colorectal cancer is the third most common cancer and the second leading cause of cancer-related deaths, with approximately 1.9 million new cases and 935,000 deaths worldwide in 2020 [1]. The prognosis of colorectal cancer varies, with disease stage representing the most important prognostic factor. Around 15–30% of patients have synchronous or metachronous metastases, and fewer than 20% of patients diagnosed with metastatic colorectal cancer survive for 5 years after diagnosis [2, 3]. However, the TNM staging system is mainly focused on tumor cells and neglects the role of the tumor microenvironment and host immune response, even though immune cell infiltration has been shown to significantly impact colorectal cancer survival [4].

Neutrophils, the most abundant immune cells in the body, act as the first line of defense in innate immunity. They are recruited into inflamed sites where they can eliminate pathogens by phagocytosis and release of antimicrobial factors. Neutrophils can also form neutrophil extracellular traps (NETs) to trap, neutralize, and kill microbes. NETs are sticky web-like extracellular structures that neutrophils produce by extruding their intracellular components, such as decondensed chromatin with cytosolic and granule proteins, in the process referred to as NETosis [5]. Important cellular events involved in NETosis are reactive oxygen species (ROS) production, activation of peptidyl-arginine deiminase 4 (PAD4), histone citrullination, and chromatin decondensation aided by neutrophil elastase (NE) and myeloperoxidase (MPO) [6, 7]. NETs can be detected by demonstrating citrullinated histone H3 (Cit-H3) [8].

Neutrophils and NETs are present in the tumor microenvironment and have been shown to play a multifaceted role in the progression of cancer [7, 9, 10]. Tumor-infiltrating neutrophils can release cytotoxic substances, directly kill cancer cells, and stimulate anti-tumor immunity. On the other hand, they can support tumor growth through mechanisms such as pro-angiogenesis activity, extracellular matrix remodeling, immune suppression, and cytokine production [11]. NET formation has been linked to increased cancer cell proliferation and metastatic processes in several cancers including colorectal cancer [12–14]. In some autoinflammatory disorders, NETosis rates positively correlate with systemic inflammation, a factor that is associated with poor prognosis in colorectal cancer [15, 16]. However, NETs have been suggested to play both pro- and anti-inflammatory roles; while they can promote inflammation, they may also limit it by degrading cytokines and chemokines [17].

The significance of NETs in colorectal cancer progression has not been clearly defined. Although tumor-infiltrating neutrophils have been more extensively studied and a recent meta-analysis reported their association with favorable survival, their impact on tumor progression is still debated [18]. Therefore, we aimed to examine the associations of tumor Cit-H3^+^ NET and CD66b^+^ granulocyte densities with prognosis, tumor-infiltrating immune cell densities, systemic inflammation markers, and other clinicopathological features in colorectal cancer. Our hypothesis was that NETs in the tumor microenvironment would be associated with poor prognosis and the activation of systemic inflammation, whereas neutrophils would be associated with better prognosis. We also hypothesized that NETs and neutrophils may be more common in mismatch repair (MMR) deficient tumors, as they are more immunogenic.

## Methods

### Study population

Our study included three cohorts (Supplementary Fig. S1). Cohort 1 consisted of a prospectively collected group of 1011 stage I–IV colorectal cancer patients who underwent surgery at Oulu University Hospital in 2006–2020 [19]. Cohort 2 included a retrospectively collected group of 1343 stage I–IV colorectal patients who had tumor resection at Central Finland Central Hospital in Jyväskylä in 2000–2015 [20]. Cohort 3 involved 77 patients enrolled in the Peri-Nutri Trial [21], all of whom were diagnosed with primary colon adenocarcinoma, considered for radical surgical treatment, and operated on at Oulu University Hospital in 2020–2024.

Patients who had received preoperative radiotherapy or chemoradiotherapy (Cohort 1: *N* = 235, Cohort 2: *N* = 243) and patients with insufficient immunohistochemistry data (Cohort 1: *N* = 16, Cohort 2: *N* = 10) were excluded from further analyses. Patients who died within 30 days after surgery (Cohort 1: *N* = 5, Cohort 2: *N* = 37) were excluded from survival analyses. Thus, 760 or 755 patients from Cohort 1 and 1090 or 1053 patients from Cohort 2 were included in the final analyses. Patients from Cohort 3 were not included in survival analyses.

In survival analyses, follow-up time was capped at 10 years. The median follow-up time for censored cases was 5.7 years (interquartile range [IQR] 3.5–8.5) in Cohort 1 and 10 years (IQR 7.3–10) in Cohort 2. Colorectal cancer-specific survival and overall survival were assessed. Colorectal cancer-specific survival was defined as the time from surgery to colorectal cancer-related death or the end of follow-up. Overall survival was defined as the time from surgery to death from any cause or the end of follow-up.

### Serum samples

From Cohort 1, preoperative peripheral vein serum samples were collected. From Cohort 3, the tumor-draining mesenteric vein blood was collected during surgery. Serum was separated by centrifugation and stored at -80°C. For Cohort 1, serum C-reactive protein (CRP) levels, albumin levels, and leukocyte differential count were measured in the laboratory of Oulu University Hospital. To assess the levels of 92 immuno-oncology proteins (Supplementary Table S1) in serum, Olink Target 96 Immuno-Oncology Panel (Olink Proteomics, Uppsala, Sweden) was used. Serum data were available for 592 patients in Cohort 1, operated between 2010 and 2020, and for 77 patients in Cohort 3. The panel utilizes Proximity Extension Assay technology, enabling high specificity and sensitivity in highly multiplexed analyses [22].

### Immunohistochemistry and image analysis

Immunohistochemistry was conducted on tissue microarrays (TMAs) that were designed to contain four cores of 1.0 mm diameter for each tumor (two from the tumor center and two from the invasive margin). *BRAF* V600E status and MMR status were defined from the TMA cores by immunohistochemistry [23, 24]. We used multiplex immunohistochemistry on TMAs to identify tumor cells (keratin), granulocytes (CD66b) and NETs (Cit-H3). The assay was performed using Leica Bond RX tissue stainer and a sequential protocol. Following deparaffinization and rehydration, antigen retrieval was carried out using BOND Epitope Retrieval Solution 2 (Leica AR9640) at 95 °C for 30 min. The tissue slides were then incubated with CD66b antibodies (BioLegend, G10F5, 1:50), and bound antibodies were visualized with the BOND Polymer Refine Red Detection kit (Leica DS9390). Antigen retrieval was repeated with BOND Epitope Retrieval Solution 1 (Leica AR9961) at 95 °C for 30 min, after which the slides were incubated with CitH3 antibodies (Cell Signaling Technology, E4O3F, 1:400), which were visualized using the BOND Polymer Refine Detection kit (Leica DS9800). In the final cycle, antigen retrieval was again performed with BOND Epitope Retrieval Solution 1 (30 min at 95 °C), and the slides were incubated with Cytokeratin antibodies (Leica, AE1/AE3, 1:100), which were visualized using the BOND Polymer Refine Detection kit (Leica DS9800), employing Green Chromogen (Leica DC9913) instead of DAB as the chromogen.

For quantitative analysis of the immunohistochemistry slides, we used QuPath (version 0.5.0), an open source bioimage analysis software for digital pathology [25]. We reviewed the TMA-cores and excluded insufficient samples from our analyses. Insufficient samples were defined as those that contained no or just a few tumor cells, were torn, or were folded beyond recognition. Random trees (RTrees) object classifier, built in QuPath to distinguish various cell types, was taught to identify tumor cells (cytokeratin staining and morphology), granulocytes (CD66b staining and morphology), and NETs (Cit-H3 staining and morphology), and to classify the remaining cells as “other”. To categorize tumor epithelial, stromal, and necrosis regions, we used the random trees (RTrees) pixel classifier built in QuPath. Areas containing whitespace, mucus, or tissue folds were excluded from the analyses. We processed the data generated with QuPath using R statistical programming (v. 4.3.3) to calculate the average densities of different cell populations for each patient. In addition to overall cell densities, we calculated the densities of Cit-H3^+^ NETs and CD66b^+^ granulocytes in the epithelial, stromal, and necrotic regions. All image analyses were performed blinded to associated data.

For Cohorts 1 and 2, densities of several additional immune cells had been evaluated using similar image analysis methods as employed in the main analysis of CD66b^+^ granulocytes and Cit-H3^+^ NETs. The densities of CD3^+^ and CD8^+^ T-cells were calculated using single-plex chromogenic immunohistochemistry [20], while the densities of CD20^+^CD79A^+^ B-cells, CD20^-^CD79A^+^ plasma-cells, as well as CD163^+^CD86^+^, CD163^-^CD83^+^, and CD163^+^CD86^-^ macrophages, were determined using multiplex immunohistochemistry coupled with digital pathology with QuPath. The details of all antibodies are presented in Supplementary Table S2. CD3-CD8 T cell density score was calculated according to the principles of Immunoscore®, based on the measurement of CD3^+^ and CD8^+^ cell densities in the tumor center and in the invasive margin [20, 26].

### Statistical analysis

For statistical analyses we used IBM SPSS Statistics program (version 29.0.0.0) or R statistical programming (v. 4.3.3). We considered findings with two-sided *P* < 0.05 to be statistically significant.

The associations between Cit-H3^+^ NET and CD66b^+^ granulocyte densities and clinicopathologic features were investigated using the Mann–Whitney U-test or Kruskal-Wallis test. Pearson’s correlation coefficients were calculated to examine the correlations of Cit-H3^+^ NET and CD66b^+^ granulocyte densities with serum inflammation markers and local immune cell densities. Adjusted beta coefficients and p values were calculated with linear regression models that included age (continuous), sex (male, female), tumor localization (colon, rectum), stage (I–II, III–IV), tumor grade (low, high), *BRAF* status (wild-type, mutant) and MMR status (proficient, deficient) as covariates. Continuous variables that were not normally distributed were logarithmically transformed. We employed the *limma* package (v. 3.58.1) in R to visualize the fold changes of mesenteric serum cytokine concentrations between patients with low and high densities (cut-point: median) of Cit-H3^+^ NETs and CD66b^+^ granulocytes as volcano plots.

We examined the associations of Cit-H3^+^ NET and CD66b^+^ granulocyte densities with cancer-specific and overall survival using Cox regression models (univariable and multivariable) and Kaplan-Meier curves. For these calculations, we divided the densities of Cit-H3^+^ NETs and CD66b^+^ granulocytes into three equal groups: low, intermediate, high. For additional analyses, Cit-H3^+^ NET and CD66b^+^ granulocyte densities were evaluated (1) as binary variables using a receiver operating characteristics (ROC) -derived optimal cut-point (maximum Youden’s index, Supplementary Fig. S2) trained in Cohort 1 and applied unchanged to Cohort 2, and (2) logarithmically transformed continuous variables.

Multivariable Cox regression models were adjusted for sex (male, female), age ( <65, 65–75, >75), year of operation (2000–2005, 2006–2010, 2011–2015, 2016–2020), tumor location (proximal colon, distal colon, rectum), disease stage (I–II, III, IV), tumor grade (low-grade, high-grade), lymphovascular invasion (negative, positive), MMR status (proficient, deficient), and *BRAF* status (wild-type, mutant). Patients with missing data on *BRAF* status (Cohort 2: *n* = 2) were included in the majority category (*BRAF* wild type) to limit the degrees of freedom.

## Results

### Cit-H3^+^ NET and CD66b^+^ granulocyte densities in relation to tumor and patient characteristics

**Fig. 1:**
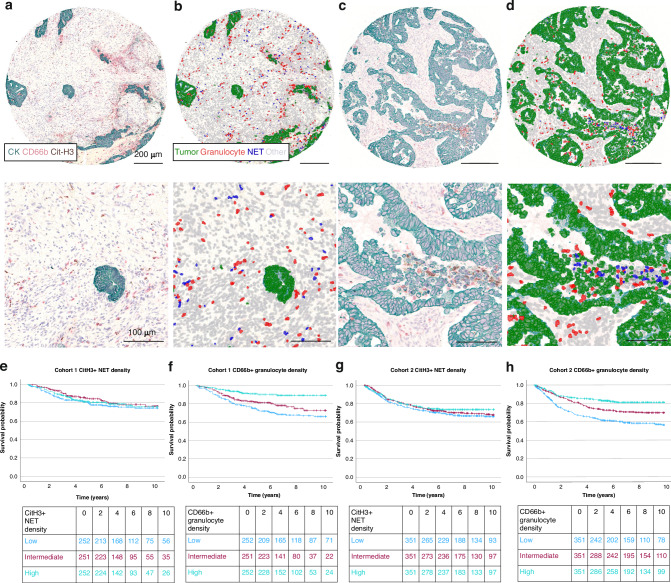
Multiplex immunohistochemistry, image analysis and Kaplan-Meier survival analyses. **a**, **c** show representative examples of the three-plex immunohistochemistry assay for the identification of neutrophil extracellular traps (NETs), granulocytes, and tumor cells. **b**, **d** show corresponding QuPath object classifier result images for the same regions. Scale bars represent 200 µm in the top row and 100 µm in the bottom row. **e–h** show relationships of citrullinated histone H3 positive neutrophil extracellular traps (**e**, **g**) and CD66b positive granulocytes (**f**, **h**) with survival in Cohorts 1 (**e**, **f**) and 2 (**g**, **h**).

Cit-H3^+^ NETs and CD66b^+^ granulocytes were successfully defined for 760 patients in Cohort 1 and 1,090 patients in Cohort 2 (Fig. 1). Their densities showed moderate positive correlation (Cohort 1: *r* = 0.545, *P* <0.001, Cohort 2: *r* = 0.551, *P* <0.001). We first studied the associations of Cit-H3^+^ NET and CD66b^+^ granulocyte densities with tumor and patient characteristics (Table 1). Our hypothesis was that increased NET densities would be associated with MMR deficiency and high stage. As for CD66b^+^ granulocytes, we hypothesized that they would be associated with MMR deficiency and favorable tumor features. In both cohorts, higher Cit-H3^+^ NET density was associated with negative lymphovascular invasion (Cohort 1: *P* = 0.003, Cohort 2: *P* <0.001) and higher tumor necrosis percentage (*P* <0.001). Additionally, higher Cit-H3^+^ NET density was associated with male sex (*P* = 0.023), MMR deficiency (*P* <0.001), and *BRAF* mutation (*P* = 0.027) in Cohort 2 but not in Cohort 1.

**Table 1:** Baseline characteristics of colorectal cancer patients according to citrullinated histone H3 positive neutrophil extracellular trap densities and CD66b positive granulocyte densities in Cohorts 1 and 2.

			Cohort 1					Cohort 2		
Characteristic	Total N	Cit-H3^+^ NET density	*P*	CD66b^+^ granulocyte density	*P*	Total N	Cit-H3^+^ NET density	*P*	CD66b^+^ granulocyte density	*P*
All cases	760 (100%)	25.3 (10.4–67.6)		150.3 (37.5–386.2)		1,090 (100%)	20.7 (7.5–55.7)		206.0 (76.1–504.1)	
Sex			0.232		0.262			0.023		0.864
Female	358 (47.1%)	27.8 (11.7–72.8)		153.1 (42.3–389.3)		538 (49.4%)	17.1 (6.7–53.2)		212.4 (71.6–512.3)	
Male	402 (52.9%)	23.6 (9.2–63.9)		147.1 (31.5–385.6)		552 (50.6%)	23.4 (8.3–59.3)		204.0 (79.6–472.6)	
Age (years)			0.940		0.546			0.094		0.008
<65	229 (30.1%)	29.6 (9.1–64.8)		134.5 (24.1–354.2)		286 (26.2%)	16.1 (6.2–53.7)		167.2 (58.5–433.3)	
65–75	279 (36.7%)	24.5 (10.7–70.7)		159.5 (37.8–391.1)		381 (35.0%)	22.3 (7.7–58.1)		200.8 (85.4–470.3)	
>75	252 (33.2%)	22.5 (11.0–67.7)		148.0 (44.4–385.8)		423 (38.8%)	23.0 (8.4–55.6)		249.8 (82.5–557.1)	
Tumor location			0.140		0.029			0.241		<0.001
Proximal colon	319 (42.0%)	24.3 (10.7–58.9)		182.8 (42.6–415.2)		531 (48.7%)	22.7 (7.9–59.1)		265.9 (97.0–578.4)	
Distal colon	204 (26.8%)	22.4 (8.6–78.5)		124.6 (30.3–286.5)		401 (36.8%)	16.0 (6.8–54.8)		169.7 (62.0–374.5)	
Rectum	237 (31.2%)	31.1 (11.2–75.5)		146.8 (33.5–394.8)		158 (14.5%)	23.9 (8.3–53.7)		185.7 (81.3–502.2)	
AJCC disease stage			0.058		<0.001			0.096		<0.001
I	174 (22.9%)	32.2 (13.0–79.0)		241.6 (87.6–561.4)		182 (16.7%)	21.5 (7.5–45.2)		287.3 (138.4–554.9)	
II	252 (33.2%)	22.4 (9.6–71.0)		150.8 (40.5–345.8)		406 (37.2%)	22.4 (8.1–61.8)		243.8 (92.1–585.9)	
III	250 (32.9%)	21.8 (8.3–59.4)		134.7 (33.8–336.4)		351 (32.2%)	21.5 (7.4–55.3)		190.7 (67.6–481.0)	
IV	84 (11.1%)	25.7 (8.9–61.1)		72.3 (17.0–212.0)		151 (13.9%)	15.1 (5.9–46.4)		105.8 (37.8–265.9)	
Tumor grade			0.311		0.109			0.510		0.009
Low-grade	650 (85.5%)	26.0 (10.5– 70.4)		147.1 (35.3–384.9)		897 (82.3%)	19.9 (7.6 – 54.8)		199.6 (71.6–463.2)	
High-grade	110 (14.5%)	21.6 (8.5–61.3)		179.5 (62.2–414.7)		193 (17.7%)	25.1 (7.0–67.0)		313.7 (95.7–616.1)	
Lymphovascular invasion			0.003		<0.001			<0.001		<0.001
No	415 (54.6%)	31.0 (12.4–77.4)		187.4 (57.6–450.4)		849 (77.9%)	23.0 (8.3–59.4)		237.5 (91.6–554.0)	
Yes	345 (45.4%)	21.2 (8.1–57.9)		99.6 (23.1–272.7)		241 (22.1%)	13.7 (5.7–43.8)		139.2 (44.4–302.8)	
Tumor necrosis percentage			<0.001		<0.001			<0.001		0.001
<3%	224 (29.5%)	19.3 (8.3–46.3)		214.1 (56.5–523.4)		251 (23.0%)	15.5 (7.3–43.8)		276.1 (123.6–599.1)	
3-39.9%	482 (63.4%)	29.8 (11.9–78.4)		137.8 (36.4–333.2)		768 (70.5%)	20.6 (7.3–50.4)		192.8 (69.5–491.1)	
≥40%	54 (7.1%)	44.0 (10.6–180.3)		76.5 (25.9–204.8)		71 (6.5%)	87.1 (26.9–266.8)		180.8 (63.5–410.7)	
Mismatch repair status			0.343		<0.001			<0.001		<0.001
MMR proficient	638 (83.9%)	24.4 (9.4–67.9)		130.7 (30.4–321.3)		925 (84.9%)	18.9 (7.1–53.7)		183.5 (67.7–423.8)	
MMR deficient	122 (16.1%)	29.5 (13.1–64.9)		346.7 (171.5–695.7)		165 (15.1%)	30.2 (11.4–87.0)		485.6 (218.1–873.6)	
*BRAF* status			0.901		<0.001			0.027		<0.001
Wild-type	653 (85.9%)	25.3 (9.9–69.1)		136.7 (31.0–348.2)		908 (83.3%)	19.3 (7.3–54.0)		186.6 (69.5–433.9)	
Mutant	107 (14.1%)	24.3 (11.5–64.0)		255.3 (105.2–634.3)		180 (16.5%)	26.9 (8.8–83.0)		393.3 (153.4–777.5)	

*P* values were calculated using the Mann–Whitney or Kruskal-Wallis test.

*AJCC* American Joint Committee on Cancer, *MMR* mismatch repair, *NET* neutrophil extracellular trap.

In both cohorts, higher CD66b^+^ granulocyte density was associated with tumor localization in the proximal colon (Cohort 1: *P* = 0.029, Cohort 2: *P* <0.001), lower disease stage (*P* <0.001), less frequent lymphovascular invasion (*P* <0.001), lower tumor necrosis percentage (Cohort 1: *P* <0.001, Cohort 2: *P* = 0.001), MMR deficiency (*P* <0.001), and *BRAF* mutation (*P* <0.001). In addition, higher CD66b^+^ granulocyte densities were associated with higher age (*P* = 0.008) and higher tumor grade (*P* = 0.009) in Cohort 2 but not in Cohort 1.

As Cit-H3^+^ NETs were frequently observed in necrotic areas, we also analyzed the association of Cit-H3^+^ NETs and CD66b^+^ granulocytes in the non-necrotic tumor stroma with various tumor characteristics (Supplementary Table S3). Higher stromal Cit-H3^+^ NET density was associated with less frequent lymphovascular invasion, lower disease stage, MMR deficiency, and *BRAF* mutation in both Cohorts 1 and 2. For stromal CD66b^+^ granulocyte densities, very similar results were observed as for densities in the overall region (including areas of necrosis).

In Cohort 3 (*N* = 77), higher Cit-H3^+^ NET densities were associated with higher age (*P* = 0.014) and MMR deficiency (*P* = 0.05). Higher CD66b^+^ granulocyte densities were associated with female sex (*P* = 0.019), higher age (*P* = 0.048), negative lymphovascular invasion (*P* = 0.027), MMR deficiency (*P* <0.001), and *BRAF* mutation (*P* = 0.005) (Supplementary Table S6).

These results show that NETs and CD66b^+^ granulocytes are more common in MMR-deficient tumors. The results also show that NETs in stroma and CD66b^+^ granulocytes are more common in low-stage tumors and tumors with no lymphovascular invasion, indicating association with favorable tumor characteristics.

### Association of Cit-H3^+^ NET and CD66b^+^ granulocyte densities with different immune cells

Next, we examined the correlations between Cit-H3^+^ NET and CD66b^+^ granulocyte densities with the densities of other tumor-infiltrating immune cells in Cohorts 1 and 2 (Table 2). We hypothesized that NETs could be associated with protumoral immune cells such as M2-like macrophages, and CD66b^+^ granulocytes with antitumoral immune cells such as cytotoxic CD8^+^ T cells. In both cohorts, Cit-H3^+^ NET densities positively correlated with the densities of CD163^-^CD86^+^ macrophages (beta = 0.211 and 0.226, *P* <0.001 and *P* <0.001 respectively) and CD163^+^CD86^+^ macrophages (beta=0.246 and 0.248, *P* <0.001 and *P* <0.001 respectively). In Cohort 2 (but not in Cohort 1), Cit-H3^+^ NET densities were also associated with the densities of CD163^+^CD86^-^ macrophages (beta = 0.163, *P* <0.001), CD3^+^ T-cells (beta = 0.075, *P* = 0.016), CD8^+^ T-cells (beta = 0.092, *P* = 0.003) and CD20-CD79A^+^ plasma cells (beta = 0.068, *P* = 0.026). CD66b^+^ granulocyte densities were positively correlated with the densities of all the analyzed immune cell types. These results underline the role of CD66b^+^ granulocytes in a complex interplay between different immune cells in colorectal cancer microenvironment and suggest a connection between NETs and macrophages.

**Table 2:** Correlations of citrullinated histone H3 positive neutrophil extracellular traps and CD66b positive granulocytes with other immune cells in Cohorts 1 and 2.

	N	Cit-H3^+^ NET density				CD66b^+^ granulocyte density			
Variable		Unadjusted		Adjusted		Unadjusted		Adjusted	
		Pearson r	*P* value	Beta	*P* value	Pearson r	P value	Beta	P value
**Cohort 1**									
CD3^+^ T cell density	759	0.068	0.060	0.044	0.26	0.185	<0.001	0.125	<0.001
CD8^+^ T cell density	759	0.086	0.018	0.074	0.059	0.285	<0.001	0.229	<0.001
CD20^+^ CD79A^+ ^B cell density	760	0.036	0.32	0.018	0.63	0.161	<0.001	0.113	0.002
CD20^-^CD79A^+^ plasma cell density	760	0.018	0.61	0.012	0.75	0.139	<0.001	0.113	0.002
CD163^+^ CD86^-^ macrophage density	759	0.072	0.049	0.063	0.099	0.149	<0.001	0.093	0.012
CD163^-^CD86^+^ macrophage density	759	0.218	<0.001	0.211	<0.001	0.277	<0.001	0.228	<0.001
CD163^+^ CD86^+^ macrophage density	759	0.240	<0.001	0.246	<0.001	0.280	<0.001	0.224	<0.001
**Cohort 2**									
CD3^+^ T cell density	1080	0.091	0.003	0.075	0.016	0.208	<0.001	0.154	<0.001
CD8^+^ T cell density	1082	0.113	<0.001	0.092	0.003	0.217	<0.001	0.154	<0.001
CD20^+ ^CD79A^+^ B cell density	1083	0.011	0.72	0.012	0.70	0.207	<0.001	0.188	<0.001
CD20^-^CD79A^+^ plasma cell density	1083	0.070	0.021	0.068	0.026	0.213	<0.001	0.191	<0.001
CD163^+^ CD86^-^ macrophage density	1085	0.173	<0.001	0.163	<0.001	0.282	<0.001	0.259	<0.001
CD163^-^CD86^+^ macrophage density	1085	0.235	<0.001	0.226	<0.001	0.329	<0.001	0.282	<0.001
CD163^+^ CD86^+^ macrophage density	1085	0.260	<0.001	0.248	<0.001	0.324	<0.001	0.279	<0.001

The adjusted beta coefficients and P values were calculated with linear regression models that included age (continuous), sex (male, female), tumor localization (colon, rectum), stage (I–II, III–IV), tumor grade (low, high), *BRAF* status (wild-type, mutant), MMR status (proficient, deficient). Continuous variables that were not normally distributed were logarithmically transformed.

*NET* neutrophil extracellular trap.

### Survival analyses

In our primary analysis, we investigated the prognostic impact of Cit-H3^+^ NET and CD66b^+^ granulocyte densities (Fig. 1, Table 3). Our hypothesis was that increased NET densities would be associated with poor outcomes, and increased CD66b^+^ granulocyte densities with better outcomes. The overall density of Cit-H3^+^ NETs was not associated with cancer-specific or overall survival. However, higher CD66b^+^ granulocyte density was associated with longer cancer-specific survival independent of disease stage, MMR status, and other factors. For cancer-specific survival, multivariable HR for high vs. low CD66b^+^ granulocyte density was 0.44 (95%CI 0.26–0.74, *P*_Trend_ = 0.004) in Cohort 1 and 0.53 (95%CI 0.38–0.73, *P*_Trend_ <0.001) in Cohort 2.

**Table 3:** Univariable and multivariable Cox regression models for cancer-specific survival and overall survival according to citrullinated histone H3 positive neutrophil extracellular trap and CD66b positive granulocyte densities in Cohorts 1 and 2.

		Colorectal cancer-specific survival			Overall survival		
	No. of cases	No. of events	Univariable HR (95% CI)	Multivariable HR (95% CI)	No. of events	Univariable HR (95% CI)	Multivariable HR (95% CI)
**Cohort 1**							
**Cit-H3**^**+**^ **NET density**							
Low	252	55	1 (referent)	1 (referent)	100	1 (referent)	1 (referent)
Intermediate	251	42	0.78 (0.52–1.16)	0.86 (0.56–1.31)	86	0.91 (0.68–1.21)	0.98 (0.73–1.33)
High	252	47	0.89 (0.60–1.31)	1.07 (0.71–1.62)	72	0.79 (0.58–1.07)	0.93 (0.67–1.28)
*P*_Trend_			0.54	0.79		0.13	0.65
**CD66b**^**+**^ **granulocyte density**							
Low	252	74	1 (referent)	1 (referent)	116	1 (referent)	1 (referent)
Intermediate	251	48	0.70 (0.48–1.00)	1.03 (0.69–1.53)	76	0.77 (0.58–1.03)	0.93 (0.68–1.27)
High	252	22	0.30 (0.19–0.49)	0.44 (0.26–0.74)	66	0.62 (0.46–0.84)	0.75 (0.54–1.05)
*P*_Trend_			<0.001	0.004		0.002	0.098
**Cohort 2**							
**Cit-H3**^**+**^ **NET density**							
Low	351	108	1 (referent)	1 (referent)	182	1 (referent)	1 (referent)
Intermediate	351	99	0.90 (0.68–1.18)	0.91 (0.69–1.20)	176	0.96 (0.78–1.18)	0.91 (0.74–1.12)
High	351	86	0.78 (0.59–1.04)	0.95 (0.71–1.26)	167	0.91 (0.74–1.12)	0.92 (0.74–1.14)
*P*_Trend_			0.092	0.68		0.37	0.46
**CD66b**^**+**^ **granulocyte density**							
Low	351	136	1 (referent)	1 (referent)	205	1 (referent)	1 (referent)
Intermediate	351	97	0.63 (0.48–0.81)	0.75 (0.58–0.98)	165	0.69 (0.56–0.85)	0.74 (0.60–0.92)
High	351	60	0.38 (0.28–0.52)	0.53 (0.38–0.73)	155	0.65 (0.53–0.80)	0.69 (0.55–0.86)
*P*_Trend_			<0.001	<0.001		<0.001	<0.001

P_trend_ values were calculated by using the three ordinal immune cell categories as continuous variables in univariable and multivariable Cox proportional hazard regression models.

Multivariable Cox proportional hazards regression models were adjusted for sex, age (<65, 65–75, >75), year of operation (2000–2005, 2006–2010, 2011–2015, 2016–2020), tumor location (proximal colon, distal colon, rectum), disease stage (I–II, III, IV), tumor grade (low-grade, high-grade), lymphovascular invasion (negative, positive), mismatch repair (MMR) status (proficient, deficient), *BRAF* status (wild-type, mutant).

*CI* confidence interval, *HR* hazard ratio, *NET* neutrophil extracellular trap.

When we restricted the analysis to granulocytes located in the tumor stroma (Supplementary Table S4), higher Cit-H3^+^ NET density was associated with longer cancer-specific survival in univariable analysis (Cohort 1: *P*_Trend_ = 0.016, Cohort 2: *P*_Trend_ =0.004), but not in multivariable models (Cohort 1: *P*_Trend_ = 0.38, Cohort 2: *P*_Trend_ = 0.82). Higher stromal CD66b+ granulocyte density was associated with improved cancer-specific survival in both univariable and multivariable models across both cohorts, with results resembling those observed for the overall region.

In sensitivity analyses, we evaluated the prognostic role of Cit-H3^+^ NETs and CD66b^+^ granulocytes as binary variables using the ROC-optimized cut-point (Supplementary Table S8) and as logarithmically transformed continuous variables (Supplementary Table S9). Consistent with primary results, CD66b^+^ granulocytes were associated with improved cancer-specific survival in both univariable and multivariable models in both cohorts, whereas Cit-H3^+^ NETs did not associate with survival.

We also compared the prognostic impact of CD66b^+^ granulocyte density and the CD3-CD8 T cell density score (based on the principles of the Immunoscore) (Supplementary Table S7). Higher CD66b^+^ granulocyte density was associated with improved cancer-specific survival independent of CD3-CD8 T cell density score in both Cohorts 1 and 2. In multivariable models adjusted for common prognostic factors, HR for high vs. low CD66b^+^ granulocyte density was 0.46 (95%CI 0.27–0.78, *P*_Trend_ = 0.011) in Cohort 1 and 0.56 (95%CI 0.40–0.79, *P*_Trend_ <0.001) in Cohort 2, while HR for high vs. low CD3-CD8 T cell density score was 0.57 (95%CI 0.31–1.05, *P*_Trend_ = 0.031) in Cohort 1 and 0.51 (95%CI 0.33–0.79, *P*_Trend_ = 0.003) in Cohort 2.

These results indicate that NET density is not a prognostic factor in CRC, whereas high CD66b^+^ granulocyte density is associated with improved survival, consistent with a potential antitumor immune role.

### Association of Cit-H3^+^ NET and CD66b^+^ granulocyte densities with systemic inflammation

Considering that NETs can activate inflammatory responses, we next assessed whether Cit-H3^+^ NET and CD66b^+^ granulocyte densities were associated with alterations in the levels of circulating inflammation markers.

**Fig. 2:**
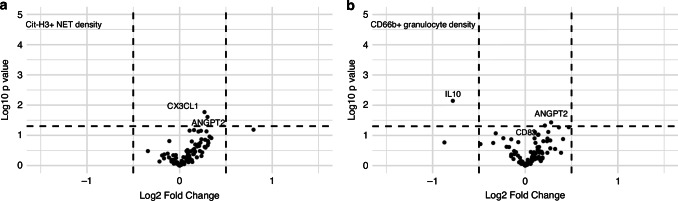
Associations of citrullinated histone H3 neutrophil extracellular traps and CD66b positive granulocytes with mesenteric venous serum inflammatory markers. The plots indicate fold changes of mesenteric venous serum inflammatory marker levels for patients with tumors that contain high vs. low (median cut-point) Cit-H3^+^ NET densities (**a**) and CD66b^+^ granulocyte densities (**b**). The analyses were based on 77 colon cancer patients (Cohort 3).

For patients in Cohort 3, mesenteric vein blood samples were collected during the operation in a unique study set-up. Mesenteric vein blood can be utilized to measure molecules before they drain to the liver and are diluted in the systemic circulation, which may give a better insight into the tumor-secreted factors, as compared to the systemic blood [27]. For 77 patients with adequate samples, we analyzed the associations between Cit-H3^+^ NET and CD66b^+^ granulocyte densities and the levels of 92 inflammation-related proteins in the mesenteric vein blood. We found four inflammation markers to have a statistically significant association with Cit-H3^+^ NET or CD66b^+^ granulocyte densities, although the fold changes between patients with low and high cell densities were modest (Fig. 2). Increased densities of Cit-H3^+^ NETs were associated with higher levels of angiopoietin 2 (ANGPT2) and C-X3-C motif chemokine ligand 1 (CX3CL1) in mesenteric blood. Increased CD66b^+^ granulocyte densities were associated with higher levels of CD83 and ANGPT2 and lower levels of interleukin 10 (IL10).

For patients in Cohort 1, preoperative peripheral serum samples had been collected, and we sought to further analyze whether Cit-H3^+^ NET and CD66b^+^ granulocyte densities were associated with circulating inflammation markers in the peripheral blood (Table 4), including blood leukocyte counts, well-established systemic inflammation markers (serum albumin, CRP, interleukin 6 [IL6], and C-X-C motif chemokine ligand 8 [CXCL8]), and the four markers (ANGPT2, CX3CL1, CD83, and IL10) that were found to correlate with Cit-H3^+^ NET or CD66b^+^ granulocyte densities in mesenteric blood samples. However, only a few weak correlations were observed. Cit-H3^+^ NET densities were negatively associated with blood lymphocyte count (beta = −0.082, *P* = 0.030) and serum albumin levels (beta =−0.097, *P* = 0.009) and positively with serum IL6 levels (beta =0.087, *P* = 0.039). CD66b^+^ granulocyte densities were inversely associated with blood neutrophil lymphocyte ratio (beta = −0.074, *P* = 0.046), blood neutrophil count (beta = −0.115, *P* = 0.002), serum CRP levels (beta = −0.094, *P* = 0.013) and serum albumin levels (beta = −0.115, *P* = 0.001).

**Table 4:** Correlations between citrullinated histone H3 positive neutrophil extracellular trap and CD66b positive granulocyte densities and inflammation markers in Cohort 1.

	N	Cit-H3^+^ NET density				CD66b^+^ granulocyte density			
Variable		Unadjusted		Adjusted		Unadjusted		Adjusted	
		Pearson r	*P* value	Beta	*P* value	Pearson r	P value	Beta	P value
Blood Neutrophil lymphocyte ratio (NLR)	715	0.012	0.75	0.017	0.65	−0.057	0.13	−0.074	0.046
Blood Neutrophil Count	715	−0.061	0.10	−0.055	0.15	−0.095	0.011	−0.115	0.002
Blood Lymphocyte Count	715	−0.081	0.030	−0.082	0.030	−0.022	0.57	−0.020	0.59
Serum CRP	704	0.041	0.27	0.058	0.14	−0.066	0.079	−0.094	0.013
Serum Albumin	750	−0.085	0.020	−0.097	0.009	−0.123	<0.001	−0.115	0.001
Serum IL6	592	0.067	0.10	0.087	0.039	−0.003	0.95	−0.009	0.82
Serum CXCL8	592	0.012	0.77	0.030	0.48	0.040	0.33	0.018	0.60
Serum CX3CL1	592	−0.013	0.76	−0.014	0.75	0.019	0.65	0.035	0.40
Serum ANGPT2	592	0.006	0.88	0.016	0.71	0.014	0.73	0.012	0.78
Serum CD83	592	0.020	0.62	0.032	0.45	0.076	0.065	0.097	0.019
Serum IL10	592	0.028	0.50	0.047	0.26	−0.002	0.95	0.021	0.60

The adjusted beta coefficients and P values were calculated with linear regression models that included age (continuous), sex (male, female), tumor localization (colon, rectum), stage (I–II, III–IV), tumor grade (low, high), *BRAF* status (wild-type, mutant), MMR status (proficient, deficient). Continuous variables that were not normally distributed were logarithmically transformed.

*ANGPT* angiopoietin, *CRP* C-reactive protein, *CXCL* chemokine (C-X-C motif) ligand, *CX3CL* chemokine (C-X3-C motif) ligand, *IL* interleukin.

Thus, our findings indicate that NET and granulocyte densities are not strongly associated with circulating cytokine levels, implying that they are unlikely to have a major role in systemic inflammation.

## Discussion

The objective of our study was to evaluate the associations of NET and granulocyte densities with tumor and patient features, local immune cells, systemic inflammation markers, and prognosis in colorectal cancer. While NET densities did not predict cancer-specific or overall survival, they positively correlated with macrophage infiltration in tumor tissue and two inflammation markers (ANGPT2 and CX3CL1) in the mesenteric blood. Higher granulocyte infiltration was associated with favorable tumor characteristics such as lower disease stage and MMR deficiency, as well as with improved cancer-specific survival independent of these factors. These results that were based on quantitative, multiplexed analysis of two large study cohorts, enlighten the role of granulocytic cells in colorectal cancer and supports their association with favorable disease course.

Elevated levels of circulating inflammatory markers, such as CRP, IL6 and CXCL8, have been reported in some colorectal cancer patients, indicating activation of systemic inflammation [16]. However, the factors driving systemic inflammation in colorectal cancer patients are not fully understood. We hypothesized that granulocytes and NETs within the tumor microenvironment might be associated with systemic inflammation. Thus, we first examined the associations between NET and granulocyte densities and 92 inflammatory markers from mesenteric blood samples. However, Cit-H3^+^ NET densities only showed weak associations with two inflammatory proteins, CX3CL1 and ANGPT2, while CD66b^+^ granulocyte densities showed weak positive correlation with mesenteric ANGPT2 and CD83 levels and weak negative correlation with IL10 levels. The associations with ANGPT2 are of potential interest, as NETs have been shown to impact angiogenesis and promote ANGPT2 release from human umbilical vein endothelial cells in gastric cancer [28, 29]. CX3CL1, a chemokine with multiple functions, has also been shown to possess pro-angiogenic effects [30]. We further analyzed whether Cit-H3^+^ NET or CD66b^+^ granulocyte densities would be associated with CX3CL1, ANGPT2, CD83, and IL10 levels in the peripheral blood, but no significant associations were found, and the correlations with other systemic inflammation markers were also weak. These findings do not strongly support a role for tumor-infiltrating NETs and granulocytes in driving systemic inflammation, although they may contribute to signaling between the tumor and the mesenteric vein blood, and potentially the liver.

Cit-H3^+^ NET densities showed a positive correlation with macrophage infiltration in both Cohorts 1 and 2. The correlation was strongest for CD86^+^ macrophages that represent macrophages polarized towards an M1-like phenotype. M1-like macrophages are generally considered to have pro-inflammatory and anti-tumor effects. NETs can damage tissues, and macrophages respond to signals from damaged tissues by initiating a pro-inflammatory program and engulfing dead cells. Macrophages can clear NETs, and it has been shown that macrophages are able to regulate NETosis in vitro [31–33]. Contrary to Cit-H3^+^ NETs, CD66b^+^ granulocyte infiltration positively correlated with the infiltration of all analyzed immune cells, supporting the interaction between different immune cells in colorectal cancer microenvironment. The strongest correlations were observed with macrophage densities, an interesting finding that aligns with evidence suggesting co-operation between macrophages and neutrophils [34], where macrophages may be able to attract neutrophils and promote neutrophil death. In contrast to their harmful effects in post-hemorrhagic shock lung inflammation, neutrophils may not be detrimental in colorectal cancer, as they could target the tumor locally.

We found no significant association between overall NET densities and survival outcomes. Increased NET densities in stroma correlated with better cancer-specific survival in univariable analyses, but not in multivariable analyses. This association might be attributed to the higher density of neutrophils in these tumors, given the positive correlation between the densities of Cit-H3^+^ NETs and CD66b^+^ granulocytes, or it could reflect antitumor actions of NETs. Although research mainly focuses on the role of NETs in supporting tumor progression, some studies have reported cytotoxic effect of NETs on tumor cells [35–37]. Also, different NETosis types may have divergent effects in cancer. A few previous studies have suggested a negative prognostic role for NETs or NET-related markers in colorectal cancer. In patients that underwent liver resection for metastatic colorectal cancer, elevated postoperative circulating NET marker levels were shown to associate with higher cancer recurrence [38]. Okamoto et al. showed that high Cit-H3 expression in tumor tissue and high serum level of MPO-DNA, a marker of systemic NET formation, correlated with poor relapse-free survival in colorectal cancer [39]. However, it was not mentioned whether necrotic areas were included in their immunohistochemical analyses, which could confound the results. Higher necrosis percentage is associated with shorter cancer-specific survival in colorectal cancer [27, 40], and according to our results Cit-H3 positivity was often found in necrotic regions. Okamoto et al. did not find significant association between Cit-H3 expression and overall survival, which is in line with our results. Thus, our findings do not support the prognostic value of intratumoral NETs in colorectal cancer, as we had hypothesized. However, we found stromal NETs to be more frequently observed in MMR deficient tumors, aligning with our hypothesis and the results by Teng et al., who showed that NET formation was augmented in patients with microsatellite instability-high compared to microsatellite stable colorectal cancer [41]. According to their study, interferon gamma upregulation had a role in driving increased NET formation. Among the major producers of interferon gamma are cytotoxic T cells, which are often densely infiltrated into MMR deficient colorectal tumors where they are activated in response to neoantigens [42, 43].

High granulocyte density was associated with longer cancer-specific survival in both Cohorts 1 and 2 independent of other prognostic parameters. Since neutrophils are the predominant CD66b^+^ granulocyte population (although eosinophils may also express CD66b [44]), this association suggests that neutrophils serve as a positive prognostic factor in colorectal cancer. To our knowledge, this study is the largest one yet to show the association between neutrophil infiltration and colorectal cancer survival. Previous studies have reported mixed findings on the prognostic value of tumor-infiltrating neutrophils, with associations to both improved and poorer colorectal cancer outcomes [45]. Zheng et al. have recently summarized these discrepancies, highlighting why methodological differences can account for mixed results [45]. For example, most studies utilizing CD66b as a neutrophil marker showed a positive prognostic effect [46–52], although a few found adverse association [53–55]. Studies recognizing neutrophils from H&E stained slides reported better [56, 57], worse [58, 59], and neutral outcomes [60, 61]. Likewise, MPO-based studies have yielded both positive and negative prognostic effects [62, 63]. Study size may also influence results; large cohorts of *N* > 1000 have more often linked neutrophils with improved prognosis [47, 48, 62]. However, one study utilizing The Cancer Genome Atlas and Gene Expression Omnibus databases (*N* = 1248) showed that relative proportion of neutrophils in colon cancer samples, estimated from bulk RNA sequencing, were associated with poor outcome [64]. A recent meta-analysis by Jiang et al., including 19 studies with 7721 patients, reported that high peritumoral neutrophil infiltration in colorectal cancer tissue was significantly associated with better cancer-specific survival, but not with overall or disease-free survival [18]. Collectively, differences in marker choice (CD66b vs. MPO vs. H&E recognition), spatial compartment (intratumoral vs. peritumoral), quantification method (quantitative multiplex vs. semi-quantitative scoring), cohort size/power, and cut-point selection likely contribute to these discrepancies.

The prognostic significance of neutrophils in colorectal cancer may be determined by their phenotypic diversity and localization within the tumor microenvironment. Tumor-associated neutrophils can be polarized along a spectrum often simplified to N1 (antitumorigenic) and N2 (protumorigenic) states [65, 66]. N1-like features include direct or antibody-dependent cytotoxicity and support of T cell responses, while N2-like functions include the promotion of tumor cell proliferation and oxidative damage, angiogenesis, and T cell suppression. A recent study that integrated multiple single cell RNA sequencing datasets identified five neutrophil clusters in colorectal cancer [67]. One of these neutrophil subpopulations was associated with worse overall survival, while others were associated with better overall survival, highlighting potential functional heterogeneity. Combining CD66b with additional markers reflecting functional states may better resolve prognostic neutrophil subtypes.

However, the large sample size of our study provides significant insight into the role of neutrophilic granulocytes in the tumor microenvironment. Neutrophils produce various cytotoxic factors and apoptosis-related ligands that directly attack tumors [68]. Although their short lifespan may limit the duration of this response, their interactions with other immune cells can trigger lasting adaptive immune responses [68]. CD66b^+^ granulocytes were associated with longer cancer-specific survival independent of CD3-CD8 T cell density score, which was calculated according to the principles of the Immunoscore®, an internationally validated T-cell-based tumor biomarker [20, 26]. This finding adds valuable information to the immune-cell-based classification of colorectal tumors.

A variety of approaches to therapeutically target neutrophils and NETs are currently under evaluation in multiple cancer types including colorectal cancer [69–71]. These include, for instance, agents targeting CXC chemokine receptor 2 (CXCR2), a major chemokine receptor involved in the trafficking of neutrophils, PAD4 inhibitors and NE inhibitors [72–74]. Despite some promising results, a notable challenge remains in selectively inhibiting tumor-promoting neutrophils while sparing or activating antitumor neutrophils. During cancer progression, neutrophils can transition between different phenotypes, and deeper understanding of the molecular mechanisms governing this polarization is essential for developing neutrophil-targeting strategies.

There are some limitations in the study. First, we analyzed NETs and granulocytes using TMAs, which may not represent immune cell infiltration in the whole tumor. However, TMAs enabled the analysis of two large cohorts using multiplex immunohistochemistry and have also been successfully utilized in numerous previous studies assessing immune cell infiltration in tumors [52, 75, 76]. Second, even though our study population was large, it was limited only to patients operated in two Finnish institutions, mostly comprising Caucasians. Therefore, the generalizability of the findings to other populations requires independent confirmation. Third, experimental studies would be required to better understand the mechanisms connecting granulocytes/NETs, systemic inflammation, and prognosis. However, research involving large human cohorts remains essential, as murine models or cell cultures cannot fully replicate the complexity of the human immune system. Because NETs have often been linked to cancer dissemination [14, 77, 78], incorporating circulating NET-related markers and assessment at metastatic sites could provide a more direct assessment of their impact on the spread and progression of colorectal cancer. Integrating spatial transcriptomics and single cell RNA sequencing could be utilized to delineate neutrophil subsets and the different tumor settings favoring their polarization.

There are some important strengths in this study. First, the use of multiplex immunohistochemistry combined with digital image analysis allowed for more precise quantification of Cit-H3^+^ NETs and CD66b^+^ granulocytes within different tumor compartments than possible using conventional qualitative analysis based on single-plex immunohistochemistry. Second, the unique mesenteric venous serum sample collection enabled us to assess circulating biomarker concentrations near the tumor. Third, a comprehensive panel of systemic inflammatory markers was analyzed, providing a more detailed evaluation of systemic inflammatory factors potentially associated with NETs/granulocytes, compared to studies that focus on only a few markers.

In conclusion, higher CD66b^+^ granulocyte densities in the colorectal cancer microenvironment are associated with improved survival independent of disease stage, MMR status, CD3-CD8 T cell density score and other tumor characteristics. While Cit-H3^+^ NETs correlate with tumor-associated macrophage densities, our results do not support their prognostic value or association with systemic inflammation. These findings underscore the potential of CD66b^+^ granulocytic cells as immune biomarkers in colorectal cancer and highlight their complex roles in the tumor immune environment. Further research on phenotypically distinct neutrophil subsets is needed to translate these findings into clinical practice. Priorities include prospective validation with standardized cut-points and assays, and biomarker-stratified immunotherapy trials that assess whether granulocyte-targeted strategies differentially benefit neutrophil-defined subgroups.

## Supplementary information

Supplementary material

## Data Availability

Data generated and/or analyzed during this study are not publicly available. The sharing of data will require approval from relevant ethics committees and/or biobanks. Further information including the procedures to obtain and access data of Finnish Biobanks are described at https://finbb.fi/en/fingenious-service.
