# Supplementary material for: Significance of tumor infiltrating granulocytes and neutrophil extracellular traps in colorectal cancer
Source: Br J Cancer. 2026 Apr 7;134(12):1692–703. doi: 10.1038/s41416-026-03409-x (PMC13226729; doi:10.1038/s41416-026-03409-x)
Supplement: Supplementary file 1 — Supplementary material [file 41416_2026_3409_MOESM1_ESM.pdf]

Significance of tumor infiltrating granulocytes and neutrophil extracellular traps in colorectal cancer

Rahkola et al.

Supplementary tables

Table S1 Biomarkers included in the Olink Immuno-Oncology Panel

UniProt ID	Gene	Protein name
O14625	CXCL11	C-X-C motif chemokine 11
P06127	CD5	T-cell surface glycoprotein CD5
Q99616	CCL13	C-C motif chemokine 13
P50591	TNFSF10	Tumor necrosis factor ligand superfamily member 10
P09038	FGF2	Fibroblast growth factor 2
Q07325	CXCL9	C-X-C motif chemokine 9
P43629	KIR3DL1	Killer cell immunoglobulin-like receptor 3DL1
P10145	CXCL8	Interleukin-8
O75509	TNFRSF21	Tumor necrosis factor receptor superfamily member 21
P07585	DCN	Decorin
O76036	NCR1	Natural cytotoxicity triggering receptor 1
P05089	ARG1	Arginase-1
P09237	MMP7	Matrilysin
P10147	CCL3	C-C motif chemokine 3
P29474	NOS3	Nitric oxide synthase, endothelial
P60568	IL2	Interleukin-2
O00182	LGALS9	Galectin-9
P35968	KDR	Vascular endothelial growth factor receptor 2
P25942	CD40	Tumor necrosis factor receptor superfamily member 5
Q8WXI7	MUC16	Mucin-16
Q14116	IL18	Interleukin-18
P20718	GZMH	Granzyme H
P01137	TGFB1	Transforming growth factor beta-1 proprotein
P09341	CXCL1	Growth-regulated alpha protein
P01730	CD4	T-cell surface glycoprotein CD4
O43557	TNFSF14	Tumor necrosis factor ligand superfamily member 14
O43508	TNFSF12	Tumor necrosis factor ligand superfamily member 12
P01127	PDGFB	Platelet-derived growth factor subunit B
Q15116	PDCD1	Programmed cell death protein 1
P48023	FASLG	Tumor necrosis factor ligand superfamily member 6
P10747	CD28	T-cell-specific surface glycoprotein CD28
Q99731	CCL19	C-C motif chemokine 19
P80075	CCL8	C-C motif chemokine 8
P13236	CCL4	C-C motif chemokine 4
P09382	LGALS1	Galectin-1
Q9NZQ7	CD274	Programmed cell death 1 ligand 1
O95760	IL33	Interleukin-33
P26842	CD27	CD27 antigen
P00813	ADA	Adenosine deaminase
P01732	CD8A	T-cell surface glycoprotein CD8 alpha chain
Q07011	TNFRSF9	Tumor necrosis factor receptor superfamily member 9
Q02763	TEK	Angiopoietin-1 receptor
P80098	CCL7	C-C motif chemokine 7
P29965	CD40LG	CD40 ligand
P01583	IL1A	Interleukin-1 alpha
Q9BZW8	CD244	Natural killer cell receptor 2B4
P01133	EGF	Pro-epidermal growth factor
Q15389	ANGPT1	Angiopoietin-1
P13232	IL7	Interleukin-7
P18627	LAG3	Lymphocyte activation gene 3 protein
Q16790	CA9	Carbonic anhydrase 9
P40933	IL15	Interleukin-15
P49763	PGF	Placenta growth factor
P05231	IL6	Interleukin-6
Q9Y653	ADGRG1	Adhesion G-protein coupled receptor G1
P13500	CCL2	C-C motif chemokine 2
O95727	CRTAM	Cytotoxic and regulatory T-cell molecule
P55773	CCL23	C-C motif chemokine 23
Q9NP84	TNFRSF12A	Tumor necrosis factor receptor superfamily member 12A
P22301	IL10	Interleukin-10
P32970	CD70	CD70 antigen

P02778	CXCL10	C-X-C motif chemokine 10
P78423	CX3CL1	Fractalkine
P09601	HMOX1	Heme oxygenase 1
P12544	GZMA	Granzyme A
P14210	HGF	Hepatocyte growth factor
P05113	IL5	Interleukin-5
P43489	TNFRSF4	Tumor necrosis factor receptor superfamily member 4
P42830	CXCL5	C-X-C motif chemokine 5
Q92583	CCL17	C-C motif chemokine 17
P21246	PTN	Pleiotrophin
Q01151	CD83	CD83 antigen
P10144	GZMB	Granzyme B
Q13241	KLRD1	Natural killer cells antigen CD94
P01375	TNF	Tumor necrosis factor
P78556	CCL20	C-C motif chemokine 20
P35225	IL13	Interleukin-13
P42701	IL12RB1	Interleukin-12 receptor subunit beta-1
P05112	IL4	Interleukin-4
O15123	ANGPT2	Angiopoietin-2
P15692	VEGFA	Vascular endothelial growth factor A
O43927	CXCL13	C-X-C motif chemokine 13
P39900	MMP12	Macrophage metalloelastase
O75144	ICOSLG	ICOS ligand
Q14790	CASP8	Caspase-8
Q9UQV4	LAMP3	Lysosome-associated membrane glycoprotein 3
P01579	IFNG	Interferon gamma
P48061	CXCL12	Stromal cell-derived factor 1
Q9BQ51	PDCD1LG2	Programmed cell death 1 ligand 2
P09603	CSF1	Macrophage colony-stimulating factor 1
P29459_P29460	IL12A_IL12B	Interleukin-12
Q29983_Q29980	MICA_MICB	MHC class I polypeptide-related sequence A and MHC class I polypeptide-related sequence B

Table S2 Antibodies

Antibody/panel	Manufacturer	Catalog number	Clone	Dilution
**Neutrophil extracellular traps**				
CD66b	BioLegend	305102	G10F5	1:50
Cit-H3	Cell Signaling Technology	97272S	E4O3F	1:400
KRT	Leica Biosystems	AE1/AE3-601-L-CE	AE1/AE3	1:100
**T cells**				
CD3	Leica Biosystems	NCL-CD3-PS1	PS1	1:50
CD8	Leica Biosystems	NCL-CD8-4B11	4B11	1:200
**B cells**				
CD20	Leica Biosystems	CD20-L26-L-CE	L26	1:75
CD79A	Cell Marque	179R-15	SP18	1:800
KRT	Leica Biosystems	AE1/AE3-601-L-CE	AE1/AE3	1:50
**Macrophages**				
CD163	Leica Biosystems	NCL-L-CD163	10D6	1:2000
CD86	Cell Signaling Technology	91882S	E2G8P	1:50
KRT	Leica Biosystems	AE1/AE3-601-L-CE	AE1/AE3	1:100

Table S3 Baseline characteristics of colorectal cancer patients according to citrullinated histone H3 positive neutrophil extracellular trap densities and CD66b positive granulocyte densities in tumor stroma in Cohorts 1 and 2.

	Cohort 1					Cohort 2				
Characteristic	Total N	Cit-H3$^+$ NET density	*P*	CD66b$^+$ granulocyte density	*P*	Total N	Cit-H3$^+$ NET density	*P*	CD66b$^+$ granulocyte density	*P*
All cases	760 (100%)	16.0 (7.2–38.1)		236.3 (65.7–557.3)		1090 (100%)	13.3 (5.3–34.4)		319.4 (120.7–708.4)	
Sex			0.125		0.167			0.393		0.868
Female	358 (47.1%)	17.8 (8.0–39.4)		269.7 (68.7–514.0)		538 (49.4%)	13.0 (5.1–32.0)		334.9 (114.5–707.5)	
Male	402 (52.9%)	14.7 (6.5–36.5)		220.4 (60.9–514.0)		552 (50.6%)	14.4 (5.5–36.6)		305.9 (131.9–710.3)	
Age (years)			0.512		0.385			0.004		0.006
<65	229 (30.1%)	18.1 (7.1–37.3)		213.0 (48.0–498.9)		286 (26.2%)	10.5 (4.5–28.0)		241.0 (84.6–681.0)	
65-75	279 (36.7%)	14.3 (7.2–36.4)		250.1 (64.4–595.4)		381 (35.0%)	14.2 (6.1–34.8)		322.3 (138.7–672.7)	
>75	252 (33.2%)	17.5 (7.3–39.1)		248.3 (77.5–573.0)		423 (38.8%)	15.7 (6.2–36.6)		369.1 (142.1–746.1)	
Tumor location			0.218		0.026			0.042		<0.001
Proximal colon	319 (42.0%)	16.6 (7.6–40.8)		298.0 (72.1–657.9)		531 (48.7%)	14.2 (5.3–40.0)		383.4 (149.8–805.2)	
Distal colon	204 (26.8%)	13.5 (6.2–34.8)		202.4 (63.6–446.8)		401 (36.8%)	11.3 (4.9–29.9)		268.4 (89.8–528.8)	
Rectum	237 (31.2%)	18.0 (7.4–37.0)		209.9 (51.9–541.5)		158 (14.5%)	15.9 (7.1–36.3)		313.8 (122.5–737.8)	
AJCC disease stage			0.001		<0.001			0.002		<0.001
I	174 (22.9%)	22.5 (9.6–47.7)		332.4 (125.3–785.6)		182 (16.7%)	14.0 (6.4–34.5)		450.8 (228.0–778.1)	
II	252 (33.2%)	16.3 (7.1–40.4)		249.9 (77.5–536.4)		406 (37.2%)	15.7 (6.3–40.3)		358.4 (155.6–809.4)	
III	250 (32.9%)	13.6 (6.4–30.9)		208.9 (57.4–479.8)		351 (32.2%)	13.1 (5.2–33.5)		277.9 (100.8–663.4)	
IV	84 (11.1%)	14.0 (6.2–32.7)		119.2 (28.4–319.0)		151 (13.9%)	9.7 (3.9–25.6)		177.3 (59.5–355.4)	
Tumor grade			0.191		0.093			0.911		0.010
Low-grade	650 (85.5%)	16.4 (7.4 – 38.9)		227.8 (63.7 – 532.9)		897 (82.3%)	13.3 (5.5 – 34.4)		302.6 (117.3 – 657.4)	
High-grade	110 (14.5%)	14.0 (5.9–30.8)		303.6 (89.4–670.2)		193 (17.7%)	13.3 (4.9–35.2)		438.9 (149.5–848.5)	
Lymphovascular invasion			<0.001		<0.001			<0.001		<0.001
No	415 (54.6%)	19.2 (8.8–43.9)		302.4 (94.9–660.1)		849 (77.9%)	15.4 (6.3–38.7)		357.7 (151.0–774.0)	
Yes	345 (45.4%)	12.8 (6.0–31.9)		162.1 (39.8–413.6)		241 (22.1%)	8.0 (4.0–21.5)		203.7 (59.2–445.2)	
Tumor necrosis percentage			0.201		<0.001			<0.001		<0.001
<3%	224 (29.5%)	18.4 (7.3–37.3)		338.3 (93.1–792.0)		251 (23.0%)	13.3 (6.5–37.9)		400.4 (198.9–878.6)	
3-39.9%	482 (63.4%)	15.6 (7.4–39.5)		219.1 (64.4–482.2)		768 (70.5%)	12.8 (5.0–30.8)		295.7 (109.8–670.4)	
≥40 %	54 (7.1%)	12.1 (5.2–26.0)		118.9 (37.9–307.3)		71 (6.5%)	31.0 (11.2–93.7)		210.6 (92.2–495.0)	
Mismatch repair status			<0.001		<0.001			<0.001		<0.001
MMR proficient	638 (83.9%)	14.6 (6.8–36.0)		203.9 (51.6–460.6)		925 (84.9%)	12.4 (5.0–30.5)		279.3 (105.6–606.3)	
MMR deficient	122 (16.1%)	25.0 (11.6–49.1)		559.4 (250.0–994.9)		165 (15.1%)	24.3 (10.1–65.2)		721.2 (329.9–1319.4)	
BRAF status			0.036		<0.001			<0.001		<0.001
Wild-type	653 (85.9%)	15.4 (7.1–36.6)		213.0 (57.2–502.1)		908 (83.3%)	12.7 (5.1–31.1)		284.4 (111.8–636.1)	
Mutant	107 (14.1%)	21.7 (9.6–47.9)		419.0 (169.7–980.6)		180 (16.5%)	22.2 (7.1–60.5)		620.7 (267.9–1128.7)	

Abbreviations: AJCC, American Joint Committee on Cancer; MMR, mismatch repair. *P* values were calculated using the Mann-Whitney or Kruskal-Wallis test.

Table S4 Univariable and multivariable Cox regression models for cancer-specific survival and overall survival according to citrullinated histone H3 positive neutrophil extracellular trap and CD66b positive granulocyte densities in tumor stroma in Cohorts 1 and 2.

		Colorectal cancer-specific survival			Overall survival		
	No. of cases	No. of events	Univariable HR (95% CI)	Multivariable HR (95% CI)	No. of events	Univariable HR (95% CI)	Multivariable HR (95% CI)
**Cohort 1**							
**Cit-H3$^+$ NET density in tumor stroma**							
Low	252	61	1 (referent)	1 (referent)	96	1 (referent)	1 (referent)
Intermediate	251	46	0.74 (0.50-1.08)	0.65 (0.44-0.97)	90	0.93 (0.70-1.24)	0.84 (0.62-1.13)
High	252	37	0.61 (0.41-0.92)	0.89 (0.58-1.35)	69	0.77 (0.57-1.05)	0.94 (0.68-1.29)
P_{Trend}			0.016	0.38		0.10	0.60
**CD66b$^+$ granulocyte density in tumor stroma**							
Low	252	75	1 (referent)	1 (referent)	116	1 (referent)	1 (referent)
Intermediate	251	44	0.62 (0.43-0.90)	0.97 (0.65-1.44)	73	0.73 (0.54-0.98)	0.89 (0.65-1.22)
High	252	25	0.34 (0.22-0.54)	0.50 (0.30-0.82)	69	0.66 (0.49-0.88)	0.78 (0.56-1.09)
P_{Trend}			<0.001	0.011		0.004	0.14
**Cohort 2**							
**Cit-H3$^+$ NET density in tumor stroma**							
Low	351	115	1 (referent)	1 (referent)	186	1 (referent)	1 (referent)
Intermediate	351	98	0.80 (0.61-1.05)	0.98 (0.75-1.30)	177	0.91 (0.74-1.11)	0.94 (0.76-1.16)
High	351	80	0.66 (0.50-0.88)	0.97 (0.71-1.31)	162	0.83 (0.67-1.03)	0.93 (0.74-1.16)
P_{Trend}			0.004	0.82		0.086	0.49
**CD66b$^+$ granulocyte density in tumor stroma**							
Low	351	134	1 (referent)	1 (referent)	198	1 (referent)	1 (referent)
Intermediate	351	96	0.65 (0.50-0.84)	0.76 (0.58-0.10)	166	0.75 (0.61-0.92)	0.80 (0.65-0.99)
High	351	63	0.42 (0.31-0.57)	0.64 (0.46-0.88)	161	0.72 (0.59-0.89)	0.80 (0.64-1.00)
P_{Trend}			<0.001	0.004		0.002	0.038

Abbreviations: CI, confidence interval; HR, hazard ratio

Multivariable Cox proportional hazards regression models were adjusted for sex, age (<65, 65–75, >75), year of operation (2000–2005, 2006–2010, 2011–2015, 2016-2020), tumor location (proximal colon, distal colon, rectum), disease stage (I–II, III, IV), tumor grade (low-grade, high-grade), lymphovascular invasion (negative, positive), mismatch repair (MMR) status (proficient, deficient), *BRAF* status (wild-type, mutant).

Table S5 Multivariable Cox regression models for cancer-specific survival in Cohorts 1 and 2.

	Cohort 1 Multivariable HR (95% CI)		Cohort 2 Multivariable HR (95% CI)	
Variable	Cit-H3$^+$ NET density	CD66b$^+$ granulocyte density	Cit-H3$^+$ NET density	CD66b$^+$ granulocyte density
Cit-H3$^+$ NET density				
Low	1 (referent)		1 (referent)	
Int	0.86 (0.56-1.31)		0.91 (0.69-1.20)	
High	1.07 (0.70-1.62)		0.95 (0.71-1.26)	
CD66b$^+$ granulocyte density				
Low		1 (referent)		1 (referent)
Int		1.03 (0.69-1.53)		0.75 (0.58-0.98)
High		0.44 (0.26-0.74)		0.53 (0.38-0.73)
Sex				
Female	1 (referent)	1 (referent)	1 (referent)	1 (referent)
Male	1.00 (0.71-1.41)	1.01 (0.71-1.42)	0.86 (0.67-1.09)	0.86 (0.68-1.10)
Age (years)				
<65	1 (referent)	1 (referent)	1 (referent)	1 (referent)
65-75	1.56 (1.02-2.39)	1.83 (1.18-2.82)	1.18 (0.87-1.59)	1.26 (0.93-1.70)
>75	2.41 (1.55-3.73)	2.66 (1.70-4.15)	1.90 (1.41-2.56)	2.03 (1.51-2.73)
Tumor location				
Proximal colon	1 (referent)	1 (referent)	1 (referent)	1 (referent)
Distal colon	1.12 (0.72-1.72)	1.19 (0.77-1.83)	0.91 (0.70-1.19)	0.91 (0.70-1.19)
Rectum	0.91 (0.58-1.41)	0.99 (0.64-1.54)	0.86 (0.59-1.24)	0.86 (0.60-1.25)
AJCC disease stage				
I-II	1 (referent)	1 (referent)	1 (referent)	1 (referent)
III	2.62 (1.56-4.39)	2.61 (1.55-4.39)	3.11 (2.26-4.28)	3.00 (2.18-4.12)
IV	18.07 (10.48-31.15)	19.24 (11.13-33.27)	18.29 (13.01-25.71)	16.83 (11.97-23.66)
Tumor grade				
Low-grade	1 (referent)	1 (referent)	1 (referent)	1 (referent)
High-grade	1.47 (0.95-2.27)	1.58 (1.02-2.44)	1.88 (1.39-2.55)	2.06 (1.52-2.80)
Lymphovascular invasion				
No	1 (referent)	1 (referent)	1 (referent)	1 (referent)
Yes	2.19 (1.38-3.48)	2.13 (1.35-3.38)	1.86 (1.45-2.39)	1.82 (1.42-2.34)
Year of operation				
2000-2005			1 (referent)	1 (referent)
2006-2010	1 (referent)	1 (referent)	0.61 (0.46-0.80)	0.58 (0.44-0.77)
2011-2015	0.86 (0.56-1.31)	0.87 (0.57-1.31)	0.46 (0.34-0.62)	0.46 (0.34-0.61)
2016-2020	0.51 (0.33-0.80)	0.57 (0.36-0.92)		
Mismatch repair status				
MMR proficient	1 (referent)	1 (referent)	1 (referent)	1 (referent)
MMR deficient	0.49 (0.24-1.03)	0.59 (0.28-1.24)	0.52 (0.31-0.86)	0.53 (0.32-0.89)
BRAF status				
Wild-type	1 (referent)	1 (referent)	1 (referent)	1 (referent)
Mutant	1.64 (0.91-2.93)	1.86 (1.04-3.32)	1.32 (0.86-2.02)	1.43 (0.93-2.20)

Abbreviations: AJCC, American Joint Committee on Cancer; MMR, mismatch repair

Table S6 Baseline characteristics of colorectal cancer patients according to citrullinated histone H3 positive neutrophil extracellular trap densities and CD66b positive granulocyte densities in Cohort 3.

Characteristic	Total N	Cit-H3$^+$ NET density	*P*	CD66b$^+$ granulocyte density	*P*
All cases	77				
Sex			0.759		0.019
Female	35	45.2 (20.6-95.9)		358.3 (138.2-709.6)	
Male	42	35.8 (19.2-94.9)		160.4 (75.0-307.8)	
Age (years)			0.014		0.048
<65	18	21.7 (9.8-37.7)		139.7 (67.0-265.2)	
65-75	28	47.3 (21.0-106.9)		176.8 (72.7-582.5)	
>75	31	60.5 (33.3-117.4)		304.7 (142.1-770.8)	
Tumor location			0.613		0.232
Proximal colon	51	44.8 (20.3-90.1)		270.8 (71.8-709.6)	
Distal colon	26	35.8 (19.7-124.9)		172.4 (108.3-283.3)	
AJCC disease stage			0.862		0.098
I	19	38.5 (20.6-95.9)		387.4 (142.1-901.1)	
II	29	38.8 (26.2-89.3)		199.7 (112.0-427.3)	
III	28	45.0 (11.9-106.9)		155.5 (68.3-562.7)	
IV	1				
Tumor grade			0.471		0.520
Low-grade	62	41.8 (23.2-94.9)		221.0 (105.1-614.8)	
High-grade	15	35.2 (6.9-111.6)		141.2 (71.8-606.1)	
Lymphovascular invasion			0.218		0.027
No	53	38.8 (27.5-92.4)		262.4 (112.0-649.3)	
Yes	24	37.8 (7.9-116.7)		139.7 (57.0-296.9)	
Mismatch repair status			0.050		<0.001
MMR proficient	57	33.6 (15.5-89.3)		164.1 (74.8-290.4)	
MMR deficient	20	61.2 (38.6-115.9)		628.7 (369.4-896.9)	
BRAF status			0.395		0.005
Wild-type	58	35.3 (19.4-91.3)		167.3 (80.6-355.1)	
Mutant	19	60.3 (27.5-111.6)		606.1 (185.9-839.4)	

Abbreviations: AJCC, American Joint Committee on Cancer; MMR, mismatch repair. *P* values were calculated using the Mann-Whitney or Kruskal-Wallis test.

Table S7. Comparison of the prognostic power of CD66b+ granulocyte density and CD3-CD8 T cell density score using Cox regression models for cancer-specific survival.

Variable	No. of cases	No. of events	Model 1 (Univariable) HR (95% CI)	Model 2 (multivariable) HR (95% CI)	Model 3 (multivariable) HR (95% CI)
**Cohort 1**					
$CD66b^+$ granulocyte density					
Low	250	73	1 (referent)	1 (referent)	1 (referent)
Intermediate	246	47	0.70 (0.48-1.01)	0.79 (0.54-1.14)	1.06 (0.71-1.58)
High	250	22	0.31 (0.19-0.49)	0.40 (0.25-0.65)	0.46 (0.27-0.78)
P_{trend}			<0.001	<0.001	0.011
CD3-CD8 T cell density score					
Low	113	52	1 (referent)	1 (referent)	1 (referent)
Intermediate	430	71	0.30 (0.21-0.43)	0.33 (0.23-0.47)	0.65 (0.43-0.98)
High	203	19	0.17 (0.10-0.29)	0.22 (0.13-0.37)	0.57 (0.31-1.05)
P_{trend}			<0.001	<0.001	0.031
**Cohort 2**					
$CD66b^+$ granulocyte density					
Low	327	124	1 (referent)	1 (referent)	1 (referent)
Intermediate	319	87	0.63 (0.48-0.83)	0.68 (0.52-0.89)	0.77 (0.58-1.02)
High	331	58	0.41 (0.30-0.55)	0.45 (0.33-0.62)	0.56 (0.40-0.79)
P_{trend}			<0.001	<0.001	<0.001
CD3-CD8 T cell density score					
Low	162	64	1 (referent)	1 (referent)	1 (referent)
Intermediate	592	171	0.69 (0.52-0.92)	0.76 (0.57-1.01)	0.79 (0.59-1.06)
High	223	34	0.34 (0.22-0.51)	0.40 (0.26-0.61)	0.51 (0.33-0.79)
P_{trend}			<0.001	<0.001	0.003

The analysis included patients from whom both $CD66b^+$ granulocyte density and CD3-CD8 T cell density score were determined. The patients who had received preoperative treatments or died within 30 days or less after the surgery were excluded, resulting 746 patients for cohort 1 and 977 patients for cohort 2.

Model 2: Cox proportional hazards regression model that included $CD66b^+$ granulocyte density and CD3-CD8 T cell density score.

Model 3: Cox proportional hazards regression model that included CD66b+ granulocyte density and CD3-CD8 T cell density score and was additionally adjusted for sex, age (<65, 65–75, >75), year of operation (2000–2005, 2006–2010, 2011–2015, 2016-2020), tumor location (proximal colon, distal colon, rectum), disease stage (I–II, III, IV), tumor grade (low-grade, high-grade), lymphovascular invasion (negative, positive), mismatch repair (MMR) status (proficient, deficient), *BRAF* status (wild-type, mutant).

P_{trend} values were calculated by using three ordinal categories of $CD66b^+$ granulocyte density and CD3-CD8 T cell density score as continuous variables in univariable and multivariable Cox proportional hazard regression models.

Abbreviations: HR, hazard ratio; CI, confidence interval

Table S8 Univariable and multivariable Cox regression models for cancer-specific survival and overall survival according to citrullinated histone H3 positive neutrophil extracellular trap and CD66b positive granulocyte densities in Cohorts 1 and 2, as binary variables using receiver operating characteristics (ROC) -derived optimal cut-points.

		Colorectal cancer-specific survival			Overall survival		
	No. of cases	No. of events	Univariable HR (95% CI)	Multivariable HR (95% CI)	No. of events	Univariable HR (95% CI)	Multivariable HR (95% CI)
**Cohort 1**							
**Cit-H3⁺ NET density**							
Low	308	69	1 (referent)	1 (referent)	125	1 (referent)	1 (referent)
High	447	75	0.77 (0.56-1.07)	0.87 (0.61-1.23)	133	0.79 (0.62-1.01)	0.86 (0.67-1.12)
P			0.12	0.43		0.059	0.27
**CD66b⁺ granulocyte density**							
Low	304	91	1 (referent)	1 (referent)	142	1 (referent)	1 (referent)
High	451	53	0.40 (0.29-0.56)	0.62 (0.43-0.91)	116	0.60 (0.47-0.77)	0.75 (0.57-0.99)
P			<0.001	0.016		<0.001	0.043
**Cohort 2**							
**Cit-H3⁺ NET density**							
Low	499	155	1 (referent)	1 (referent)	255	1 (referent)	1 (referent)
High	554	138	0.79 (0.63-1.00)	0.92 (0.72-1.16)	270	0.95 (0.80-1.12)	0.96 (0.80-1.14)
P			0.045	0.47		0.52	0.64
**CD66b⁺ granulocyte density**							
Low	311	119	1 (referent)	1 (referent)	180	1 (referent)	1 (referent)
High	742	174	0.55 (0.44-0.70)	0.69 (0.54-0.88)	345	0.71 (0.60-0.85)	0.75 (0.62-0.91)
P			<0.001	0.003		<0.001	0.003

Abbreviations: CI, confidence interval; HR, hazard ratio
Multivariable Cox proportional hazards regression models were adjusted for sex, age (<65, 65–75, >75), year of operation (2000–2005, 2006–2010, 2011–2015, 2016-2020), tumor location (proximal colon, distal colon, rectum), disease stage (I–II, III, IV), tumor grade (low-grade, high-grade), lymphovascular invasion (negative, positive), mismatch repair (MMR) status (proficient, deficient), *BRAF* status (wild-type, mutant).

The optimal cut-points were derived in Cohort 1 (maximum Youden's index) and applied unchanged to Cohort 2.

Table S9 Univariable and multivariable Cox regression models for cancer-specific survival and overall survival according to logarithmically transformed continuous citrullinated histone H3 positive neutrophil extracellular trap and CD66b positive granulocyte densities in Cohorts 1 and 2.

	Colorectal cancer-specific survival		Overall survival	
	Univariable HR (95% CI)	Multivariable HR (95% CI)	Univariable HR (95% CI)	Multivariable HR (95% CI)
**Cohort 1**				
**Cit-H3$^+$ NET density**	0.94 (0.70-1.24)	1.08 (0.78-1.50)	0.85 (0.69-1.05)	0.94 (0.74-1.19)
P	0.64	0.65	0.13	0.58
**CD66b$^+$ granulocyte density**	0.63 (0.52-0.77)	0.73 (0.57-0.93)	0.80 (0.69-0.93)	0.86 (0.72-1.02)
P	<0.001	0.010	0.004	0.079
**Cohort 2**				
**Cit-H3$^+$ NET density**	0.86 (0.70-1.04)	1.00 (0.82-1.23)	0.94 (0.81-1.09)	0.98 (0.84-1.14)
P	0.12	0.98	0.42	0.77
**CD66b$^+$ granulocyte density**	0.54 (0.44-0.65)	0.74 (0.60-0.90)	0.74 (0.64-0.85)	0.81 (0.69-0.95)
P	<0.001	0.003	<0.001	0.008

Abbreviations: CI, confidence interval; HR, hazard ratio
Multivariable Cox proportional hazards regression models were adjusted for sex, age (<65, 65–75, >75), year of operation (2000–2005, 2006–2010, 2011–2015, 2016-2020), tumor location (proximal colon, distal colon, rectum), disease stage (I–II, III, IV), tumor grade (low-grade, high-grade), lymphovascular invasion (negative, positive), mismatch repair (MMR) status (proficient, deficient), *BRAF* status (wild-type, mutant).

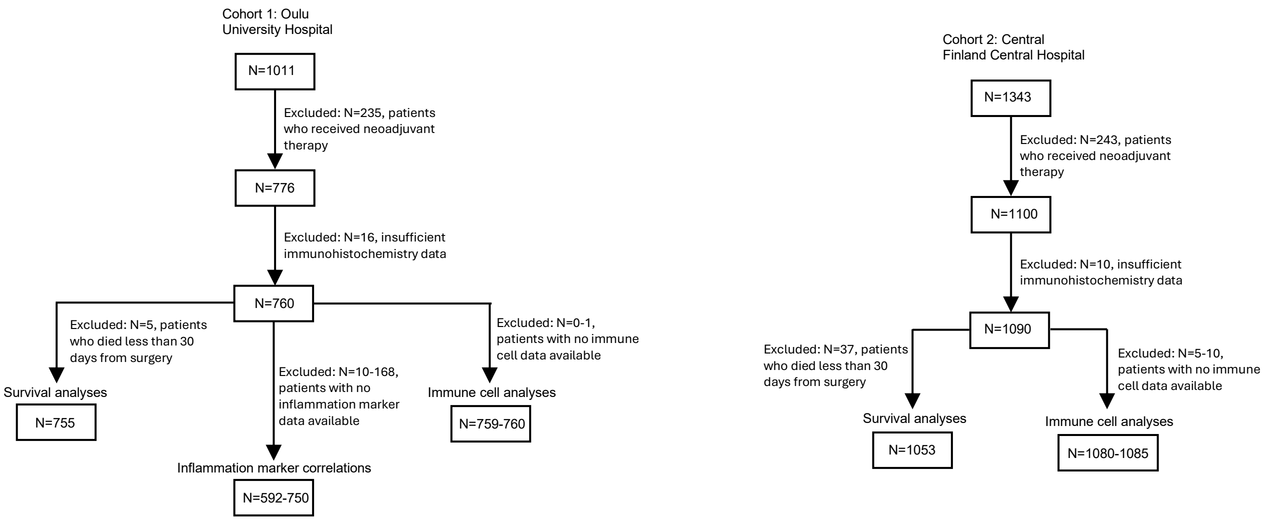

Figure S1. Flow-charts of Cohorts 1 and 2 analyzed in the study.

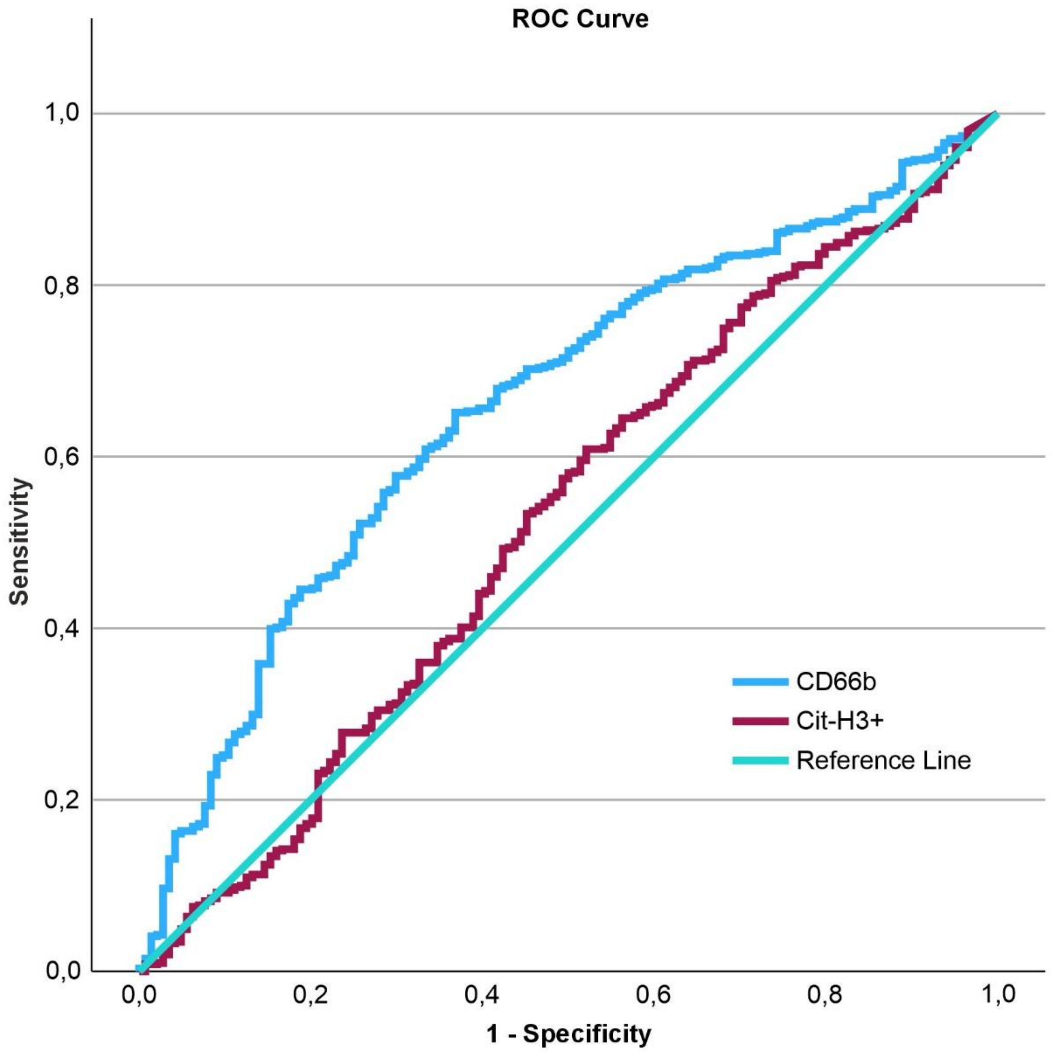

Figure S2. Receiver operating characteristics (ROC) curves for citrullinated histone H3 positive neutrophil extracellular traps and CD66b positive granulocyte densities in predicting cancer-specific survival in Cohort 1.
